# Dose-dense TPF induction chemotherapy for locally advanced head and neck cancer: a phase II study

**DOI:** 10.1186/s12885-020-07347-6

**Published:** 2020-09-01

**Authors:** Ching-Yun Hsieh, Ming-Yuh Lein, Shih-Neng Yang, Yao-Ching Wang, Yin-Jun Lin, Chen-Yuan Lin, Chun-Hung Hua, Ming-Hsul Tsai, Ching-Chan Lin

**Affiliations:** 1 Division of Hematology and Oncology, Department of internal medicine, China Medical University Hospital, China Medical University, 2 Yude Rd, North District, Taichung, 404 Taiwan; 2 Division of Radiation Oncology, China Medical University Hospital, China Medical University, Taichung, 404 Taiwan; 3 Department of Otorhinolaryngology, China Medical University Hospital, China Medical University, Taichung, 404 Taiwan; 4 Graduate Institute of Biomedical Sciences, China Medical University, Taichung, 404 Taiwan

**Keywords:** Head and neck cancer, Docetaxel, Cisplatin, Fluorouracil, Dose-dense chemotherapy, Induction chemotherapy

## Abstract

**Background:**

Phase 3 studies suggest that induction chemotherapy (ICT) of cisplatin and 5-fluorouracil plus docetaxel (TPF) is effective but toxic for patients with squamous-cell carcinoma of the head and neck (SCCHN). Dose-dense chemotherapy may yield favorable outcomes compared with standard-dose chemotherapy, yet the optimal induction regimen remains undefined. We assessed the efficacy and tolerability of biweekly dose-dense TPF ICT in patients with SCCHN.

**Methods:**

In this prospective phase II study, We enrolled patients with stage III/IV (AJCC 7th edition) unresectable squamous cell carcinoma of head and neck cancer. Patients received dose-dense TPF (ddTPF) with cisplatin and docetaxel 50 mg/m2 on day 1, leucovorin 250 mg/m2 on day1, followed by 48-h continuous infusion of 2500 mg/m2 of 5-fluorouracil on day 1 and 2, every 2 weeks for 6 cycles followed by radiotherapy. The primary endpoint was the response rate (RR) after ICT.

**Results:**

Fifty-eight patients were enrolled from June 2014 to September 2015. Overall RR after ICT was 89.6% [complete response (CR), 31%; partial response (PR), 58.6%]. Grade 3/4 neutropenia, mucositis, and diarrhea incidences were 25.9, 1.7, and 1.7%, respectively. 94.8% of patients completed all treatment courses of ICT without dose reduction. The 3-year overall survival (OS) was 54.3% (95%CI: 39.7 to 66.8%) and progression-free survival (PFS) was 34.3% (95%CI: 22.0 to 46.9%). Multivariate analysis showed that CR after ICT is an independent prognostic factor for OS and PFS.

**Conclusions:**

Six cycles of ddTPF is an active, well-tolerated induction regimen for patients with SCCHN. The presence of CR after ICT predicted long-term survival.

**Trial registration:**

ClinicalTrials.gov Identifier: NCT04397341, May 21, 2020, retrospectively registered.

## Background

Chemotherapy combined with radiotherapy (RT) is crucial for patients with locally advanced squamous cell carcinoma of the head and neck (SCCHN) who are inoperable or who require organ preservation. It includes concurrent chemoradiotherapy (CRT) and induction chemotherapy (ICT) followed by RT or CRT [1, 2]. The most common ICT regimen included cisplatin plus 5-fluorouracil (PF) [3]. However, the PF ICT group was inferior to the CRT group in locoregional control and event-free survival [4]. To improve the efficacy of ICT, a revolutionary treatment strategy in the recent decade has been the addition of docetaxel to PF ICT. Two phase III trials confirmed that TPF ICT provides a significant survival advantage compared with PF ICT [5, 6]. However, whether TPF ICT plus CRT may also obtain better survival outcomes than the standard CRT is controversial. Two phase III trials showed that TPF ICT plus CRT did not reveal a statistically significant survival benefit compared with CRT alone [7, 8]. Furthermore, only a phase III trial of TPF ICT reached the OS benefit compared with the no-induction group [9]. The most significant clinical concern is the more severe adverse events especially of grade 3/4 neutropenia, and TPF-related deaths, which may compromise treatment efficacy [10].

In the TPF regimen, 75 mg/m^2^ of docetaxel every 3 weeks is a widespread prescription based on the TAX 323 study [5]. However, the toxicities of triweekly docetaxel have attracted a lot of worries especially for the elderly patients and the Asian population [11, 12]. In a prospective pharmacokinetic study of 75 mg/m2 docetaxel every 3 weeks for patients with solid tumors, 63% of elderly patients experienced grade 4 neutropenia compared to 30% of younger patients [12]. The analysis from published data of phase II and III clinical trials showed that a higher incidence of docetaxel-induced grade 3/4 neutropenia in Asian clinical studies than the non-Asian studies (odds ratio 19.0, [13]). In addition, hematologic toxicity still occurs in these patients even with the reduced dose of triweekly TPF [14, 15].

Therefore, although TPF regimen is an effective regimen for patients with SCCHN, the optimal scheduling remains unclear. One of the promising alternatives is the dose-dense chemotherapy regimen, which can be reached by the same dose intensity administered at a shorter time-interval, for instance at a biweekly interval. Dose-dense chemotherapy does not mean greater toxicity. In fact, the experience from the C9741 trial for patients with breast cancer showed that more dose-dense administration was associated with less neutropenia and less need for hospitalization due to febrile neutropenia [16].

In terms of SCCHN, to the best of our knowledge, only one retrospective study reported the biweekly modified TPF chemotherapy was safe and was as effective as triweekly TPF [17]. However, the decreased dose-intensity of cisplatin and docetaxel in this study made the interpretation of the study difficult when compared to the “classical “triweekly TPF regimen. Additionally, the clinical trials in bladder and breast cancers have shown that the benefit of a dose-dense approach in the neoadjuvant setting, but the studies had not been explored in SCCHN [18]. Therefore, we designed a phase II study to evaluate the efficacy and toxicity of the biweekly dose-dense TPF.

## Methods

### Patients

This prospective single-arm phase II trial enrolled the patients with newly diagnosed locally advanced SCCHN. The study was conducted in accordance with the World Medical Association Declaration of Helsinki (version 2002) and was approved by the China Medical University Hospital Review Board (CMUH103-REC2–038). Patients provided IRB-approved, protocol-specific written informed consent prior to initiating therapy for study-specific treatment and inclusion in the present study. The study was retrospectively registered in ClinicalTrials.gov (Identifier: NCT04397341).

The inclusion criteria were as follows: (1) histopathologically confirmed diagnosis of AJCC 7th edition stage 3 or 4 unresectable squamous cell carcinoma of the oral cavity, larynx, oropharynx, or hypopharynx; (2) age > 20 years; (3) ECOG performance status, 0, 1, or 2 at study entry; and (4) adequate organ function. The exclusion criteria were as follows: (1) previous chemotherapy or RT for SCCHN, (2) previous or concurrent malignancy, and (3) peripheral neuropathy > grade 2. Additionally, patients were defined as having an unsuitable condition for radical surgery after the evaluation of a multidisciplinary team. The inoperability criteria included very advanced T stage (T4b) (tumor invasion into the cervical vertebra, carotid artery, or fixed lymph nodes), risk of significant postoperative dysfunction, and low surgical curability (T3–T4, N2–N3).

### Treatment

Patients received ICT after enrollment. The chemotherapy regimen was delivered with docetaxel 50 mg/m2 on day 1, cisplatin 50 mg/m2 on day 1 and 5-fluorouracil 2500 mg/m2 by continuous infusion over 46 h on Day 1–2. Moreover, the treatment was administered every 14 days for 6 cycles or until disease progression or intolerant treatment toxicity. In our study, primary G-CSF support was not allowed, but G-CSF administration can be used when the patients had febrile neutropenia or grade 4 neutropenia.

The dose modification was allowed, which was according to the greatest degree of toxicity that was graded by NCI-CTCAE Version 4.03. Also, the dose modification of study treatment could be according to the investigator’s experiences.

Radiotherapy was administered using the sequential Intensity-Modulated Radiotherapy (IMRT) for 1.8–2.0Gy per fraction with five daily fractions per week for 7 weeks. Two clinical target volumes (CTVs) were designed for risk stratification: CTV1, encompassing the gross tumor volume (GTV) of a primary tumor, metastatic lymph nodes, and regions adjacent to the GTV; and CTV2, encompassing the ipsilateral or contralateral N0 regions at risk of microscopic tumor. During the first course, a dose of 50.4–54.0Gy was included in CTV1 and CTV2. During the second course, a boost of 16.2–21.6Gy was included in CTV1. The median cumulative dose of 70.2 and 54.0Gy was achieved in CTV1 and CTV2, respectively. Concurrent chemotherapy with weekly cisplatin 35 mg/m2 was recommended during the period of radiotherapy. However, it is allowed to give radiotherapy alone or substitute cetuximab for cisplatin as the radiosensitizer for the patients not suitable for concurrent chemoradiotherapy by the assessment and discretion of the attending physician. Cetuximab was used at a 250 mg/m2 on a weekly basis (400 mg/m2 as the initial dose) for seven times.

### Study design, end point definition, and statistical considerations

This was a phase II, single-center, prospective clinical trial to evaluate the induction chemotherapy (ICT) with a biweekly dose-dense TPF regimen for SCCNHN patients. The primary endpoint of the study was the response rate (RR) of ICT, while the secondary ones included response rate (RR) of the total treatment, progression-free survival (PFS), overall survival (OS), and toxicity and safety of the treatment. “The response assessment of ICT (the primary endpoint) was performed using CT scan at the end of treatment according to the RECIST 1.1 criteria. Positron emission tomography (PET)/CT scan was also performed to evaluate the response according to the European Organization for Research and Treatment of Cancer (EORTC) 1999 criteria.” In addition, only when CT scans failed to reveal an obvious primary, PET/CT scan was used to confirm the complete response. The response assessment of the total treatment was performed at 10–12 weeks since the end of radiotherapy with CT scan, and the response assessment was confirmed by repeat CT scan at an interval of 6–8 weeks. Then, the CT scan was performed every 3–4 months in the first 2 years and every 6–7 months until progression. The PFS was defined as the time from the study registration date to the first day of disease progression at any site or of death by any cause. The OS was defined as the time from the date of study registration to the date of death, or the last confirmed survival date. The toxicity was recorded by the investigators using the National Cancer Institute Common Toxicity Criteria scale version 4.03 (NCI-CTCAE Version 4.03). Toxicities were assessed at each weekly visit during ICT and at the end of treatment.

At the time of study design, the response rate of standard triweekly TPF regimen was reported to be 68 and 72%, respectively [5, 6]. Therefore, the study was conducted using Simon’s optimal two-stage phase II design based on response rate for sample-size calculation. A sample size of 35 patients was required to accept the hypothesis that the true response rate was greater than 90% with a 90% power and to reject the hypothesis that the response rate was less than 70% with 5% significance. Initially, we planned to enroll 15 patients in the first stage. If 11 or more responses were observed, we planned to continue to the second stage for a total of 35 patients in the analysis. Assuming a dropout rate of 10%, the total number of enrolled patients needed was calculated to be 40. If the endpoints were achieved, we will extend the number of enrolled patients to 60 for translation study. The translational study aims to study the prognostic significance of the metabolic parameters of PET in tumour response to ICT.

Kaplan-Meier survival analysis was used to estimate survival endpoints, and the log-rank test was used to compare the differences between curves. All tests are two-sided, and a *p*-value < 0.05 was considered to indicate a statistically significant difference. Multivariate analysis was carried out using a Cox proportional hazards regression model. The age was classified into > 50 and ≦50 years old using the receiver operating characteristic (ROC) curve.

Statistical analysis was performed with the SPSS software (version 17.0; SPSS, Chicago, IL) and EZR version 1.32. software [19].

## Results

### Patients

**Fig. 1:**
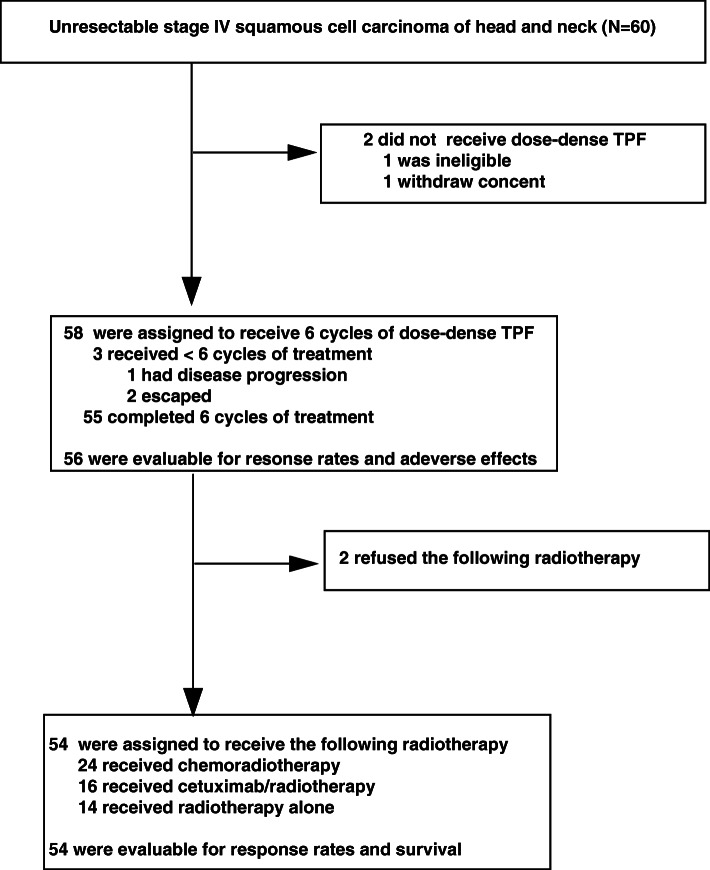
Enrollment and outcomes

At the first stage of the study, thirteen of 15 patients achieved better than stable disease after ICT treatment. As a consequence, twenty-five patients were further enrolled in the second stage. Overall thirty-five of 40 patients were evaluated to be responders to ICT. The response rate was estimated to be 87.5%. Finally, a total of 60 patients were enrolled in the study from June 2014 to September 2015. Among the 60 patients, only fifty-eight patients were assessable because one patient was excluded because of ineligibility and one withdrew the consent (Fig. 1). The clinical characteristics of the patients were demonstrated in Table 1. The median follow-up time was 31.8 months (from 1.4 to 43.6 months). The median age is 53 years old (from 28 to 69 years old). All of them had stage IV SCCHN (IVA, 60.3%; IVB, 39.7%, AJCC 7th edition). The primary site of oral cavity/oropharynx/hypopharynx was 31.0%/43.1%/25.9%. Initially, all of the patients were recruited by AJCC 7th edition, but the stage status was analyzed later according to AJCC 8th edition. Six of 25 (24%) patients with oropharyngeal cancer (OPC) were considered HPV-associated by the immunohistochemical stain of p16, and 19 (76%) patients were HPV negative. Most patients reported the history of drinking and smoking.

**Table 1:** Clinical characteristics of the patients

Characteristics *N* = 58	Number	percentage
Age,median (range): 53 (28–69)		
< 50	16	27.6
50–65	40	69.0
≥ 65	2	3.4
Gender		
Male	54	93.1
Female	4	6.9
ECOG performance status		
0	37	63.8
1	21	36.2
2	0	0
Smoking		
Yes	49	84.5
No	9	15.5
Alcohol drinking		
Yes	44	75.9
No	14	24.1
Betal nut chewing		
Yes	43	74.1
No	15	25.9
Stage (AJCC 8th edition)		
III (HPV+ oropharynx)	6	10.3
IVa	13	22.4
IVb	39	67.3
T		
1,2	13	22.4
3	9	15.5
4a	13	22.4
4b	23	39.7
N		
0	5	8.6
2 (HPV+ oropharynx)	3	5.2
2b	14	24.1
2c	11	19.0
3	25	43.1
Site		
Oral cavity	18	31.0
Oropharynx	25	43.1
HPV associated	6	10.4
HPV negative	19	32.7
Hypopharynx	15	25.9
Neck dissection after ICT		
Yes	8	13.8
No	50	86.2

### Efficacy and outcome

All the 58 patients received ICT with biweekly TPF. Fifty-five of 58 (94.8%) patients completed all treatment courses of ICT. Three patients did not complete the 6-cycle treatment including two who escaped during ICT and the other one had early disease progression. The mean accumulative dose of docetaxel, cisplatin and 5-fluorouracil was 288 mg/m2, 288 mg/m2, and 14,474 mg/m2, respectively. Therefore, the relative dose intensity was 96, 96 and 96.5%, respectively. No dose reduction occurred in all of the patients administered with ICT. Four patients had dose delay of more than 7 days because of infection. The response rates (RR) of ICT was shown in Table 2. On CT scan, 12 of 58 (20.7%) patients achieved the complete response, but on PET/CT scan, 18 (31%) had the metabolic CR (CR). Three patients achieved a stable disease (SD) and 34 patients had a partial response (PR). The overall RR of ICT was 89.7%. All of the patients received full-dose ICT, and none had the reduced dose of docetaxel, cisplatin, or 5-fluorouracil.

**Table 2:** Response to induction chemotherapy and to total treatment of induction therapy plus radiotherapy

	Number	Percentage
Response of Induction chemotherapy (*N* = 58)		
Overall RR	52	89.7%
CR	18	31.0%
PR	34	58.6%
SD	3	5.2%
PD	3	5.2%
Response of ICT plus RT (*N* = 54)		
Overall RR	42	77.8%
CR	37	68.5%
PR	5	9.3%
SD	6	11.1%
PD	6	11.1%

*CR* complete response, *ICT* induction chemotherapy, *PR* partial response, *PD* progressive disease, *RR* response rate, *RT* radiotherapy, *SD* stable disease

After ICT, 2 patients refused the following radiotherapy. Twenty-four patients received chemoradiotherapy, 16 patients received cetuximab/RT, and 14 patients received RT alone. Therefore, a total of 54 patients is evaluable for treatment response after radiotherapy. Three of 54 (5.5%) cannot complete the RT program because that one patient had severe pneumonia, another developed ischemic stroke during the treatment, and one escaped from the treatment protocol. After the CRT or RT process, 37 (68.5%) patients achieved CR, 5 (9.3%) had a PR, 6 (11.1%) had a stable disease, and 6 (11.1%) had disease progression. The overall RR of the total treatment was 77.8%.

**Fig. 2:**
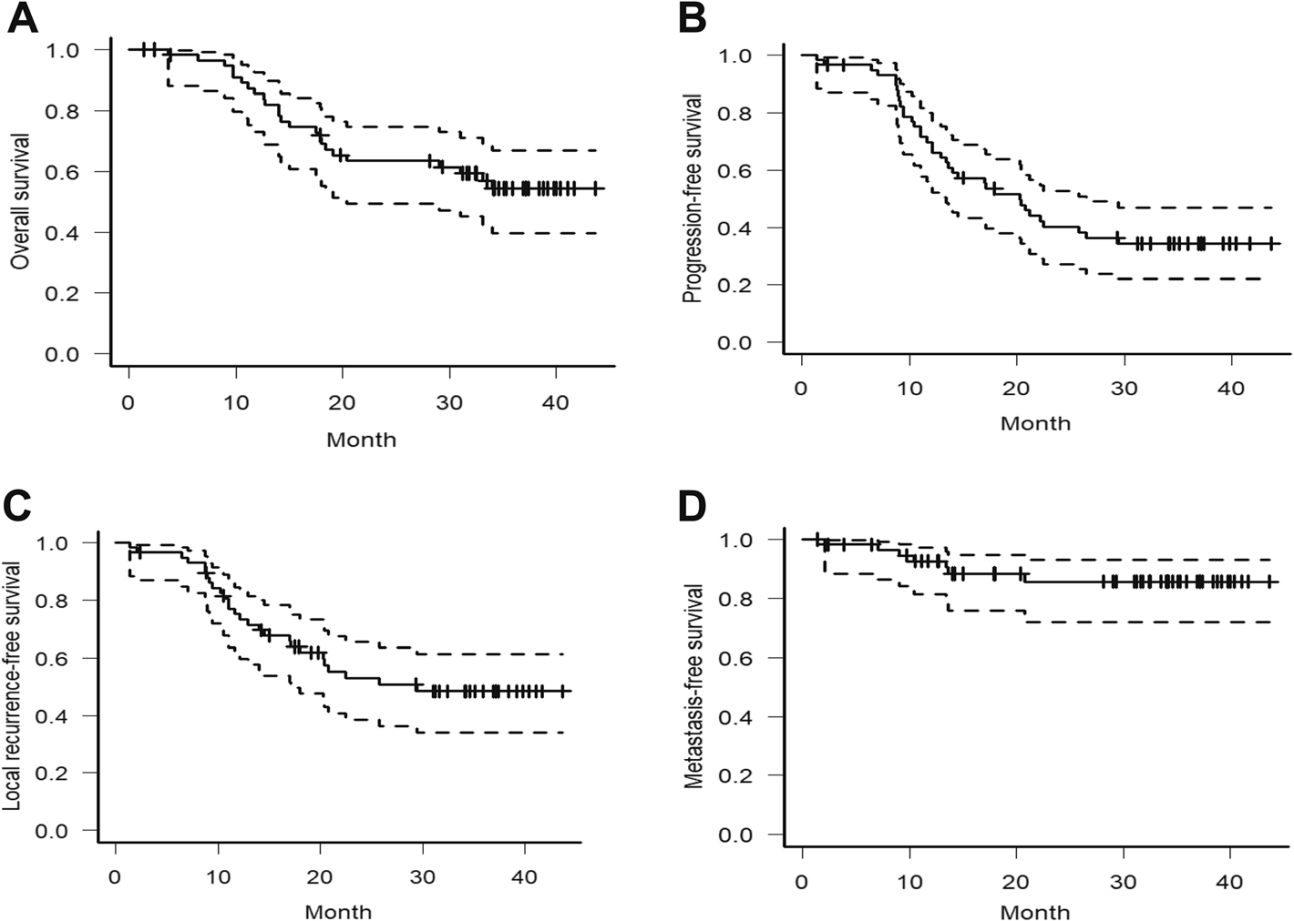
Survival analsysis. **a** Overall survival. **b** Progression-free survival. **c** Local recurrence-free survival. **d** Metastasis-free survival

The 3-year OS and PFS were 56.3% (95%CI, 41.6–68.6%) and 34.3%, (95%CI, 22.0–46.9%), respectively, as seen in Fig. 2a and b. The 3-year local-recurrence free survival was 48.4% (95%CI, 34.1–46.9%) and the 3-year metastasis free-survival was 85.6% (95%CI, 71.9–93.0%), as seen in Fig. 2c and d.

**Fig. 3:**
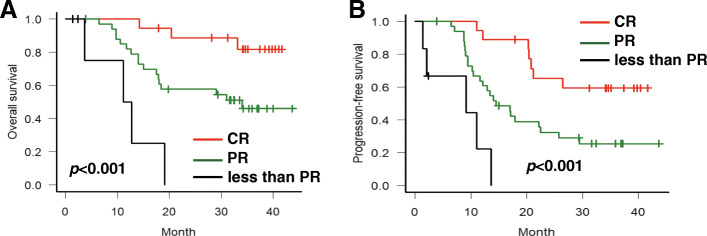
Survival analysis stratified by response rate (RR) of ICT. **a** Overall survival. **b** Progression-free survival. CR: complete response; PR: partial response. ICT: induction chemotherapy

We also analyzed the association between tumour response (RR) of ICT and the survival outcomes of the patients. The Kaplan–Meier analysis showed that the patients with complete response had superior survival to those with partial response and nonresponders. The 3-year OS and PFS were 81.7% (95%CI, 53.1–93.8%) and 59.3% (95%CI,33.0–78.1%) in patients with complete response (*p* value < 0.001) and 45.9% (95%CI,27.4–62.6%) and 25.5% (95%CI,11.9–41.4%) in patients with partial response (*p* value < 0.001), respectively, as seen in Fig. 3. All patients with the response less than PR passed away within 20 months. We also performed the multivariate analysis to consider other variables, including age, stage, primary site, and smoking status. We did not include HPV status in Cox regression analysis because only 6 patients were HPV-associated. The results showed that response status after ICT was the only significant prognostic factor for OS and PFS (Table S1).

### Treatment-related adverse events

The treatment-related adverse events during ICT are shown in Table 3. During this, mucositis was the most common event (33/58, 56.9%), but only one patient suffered from grade 3 mucositis. In grade 3/4 adverse events, 15 patients (25.9%) had neutropenia, and 6 (10.3%) developed febrile neutropenia. Also, 11 patients developed infection; however, no patients died during the process.

**Table 3:** Adverse effects of treatment

NICIC CTG grade	Grade 1,2	Grade 3,4
During induction chemotherapy (*n* = 58)		
anemia	5 (13.79%)	1 (1.72%)
neutropenia	5 (13.79%)	15 (25.86%)
thrombocytopenia	0	0
alopecia	0	58 (100%)
Weight loss	3 (5.17%)	0
Hand foot syndrome	28 (48.27%)	0
Mucositis/stomatitis	33 (56.89%)	1 (1.72%)
Febrile neutropenia	0	6 (10.34%)
Acute kidney injury	17 (29.31%)	0
Hyponatremia	19 (32.75%)	18 (31.03%)
Hypokalemia	20 (34.48%)	5 (13.79%)
nausea	12 (20.68%)	0
vomiting	1 (1.72%)	0
Diarrhea	10 (17.24%)	1 (1.72%)
Hearing loss	10 (17.24%)	0
Lethargy	4 (6.89%)	1 (1.72%)
Peripheral neuropathy	29 (50.00%)	0

## Discussion

Our study showed that biweekly dose-dense TPF ICT in locally advanced SCCHN had a promising treatment response. The overall response rate and complete response rate were 89.7 and 31%, respectively. The 3-year OS and PFS were 56.3 and 34.3%, respectively. Compared to the data from TAX323 and TAX324 trials, standard triweekly TPF ICT regimen achieved 68 and 72% post-induction RR, respectively [5, 6]. The 3-year OS and PFS for patients in TAX 323 were 37 and 17%, and those in TAX 324 were 62.0 and 49.0%, respectively [5, 6]. Particularly, 25.9% of the grade 3–4 neutropenia in our study is markedly lower than previously reported. For example, TAX323 trial reported 76.9% of the grade 3–4 neutropenia, and TAX324 trial reported 83% of the grade 3–4 neutropenia [5, 6].

Toxicity is a major issue of ICT, with up to 6% toxic deaths and 11% of febrile neutropenia [4, 20]. Some studies had tried to find fewer toxic regimens by reducing chemotherapy dose [21, 22]. HM. Wang et al. reduced the dose to 90% of the original TPF dosage (docetaxel 67.5 mg/m2 on Day 1, cisplatin 67.5 mg/m2 on Day 1 and 5FU 675 mg/m2 on Day 1–5) as the induction treatment. The grade 3–4 neutropenia was 35%, and the overall response rate was 73.1% after induction therapy [21]. A retrospective study from J Fayette et al. in France used the modified TPF regimen of docetaxel and cisplatin at 40 mg/2 on day 1 and a bolus of 5FU at 400 mg/m2, then 1000 mg/m2 on Day 1–2 every 2 weeks. The authors reported 10% of febrile neutropenia, 83% of overall response rate and 19% of complete response [22]. Notably, the regimen adopted by J Fayette et al. was similar to that our study used, such as biweekly administration, and 48-h infusion of high-dose 5FU. Biweekly administration of docetaxel might be better tolerated due to the reduced peak drug concentrations [23]. Data from colon cancer and gastric cancer had shown that biweekly 48 h infusion of 5FU can increase the response rate with fewer side effects of myelotoxicity [24, 25]. However, the inherent bias of the retrospective study, the heterogenous post-ICT treatment (including surgery and radiotherapy) and the reduced dose intensity of cisplatin and docetaxel compared to the standard TPF regimen made the interpretation of the results difficult.

Dose-Dense Chemotherapy aims to achieve maximum tumor kill by shortening the interval of chemotherapy delivery. Several clinical trials and metastasis from breast cancer, bladder cancer, and lymphoma had revealed that dose-dense regimen improved response rate and survival outcomes [26, 27]. However, the associated studies in head and neck cancer are scarce. Our TPF regimen included 50, 50, 2500, and 250 mg/m^2^ of docetaxel, cisplatin, 5-fluorouracil, and leucovorin, respectively. We maintained the average dose intensity of the traditional TPF, with less toxicity than triweekly TPF and similar rates of neutropenia (25.86%) compared with the weekly TPF regimen. Additionally, 94.8% of patients completed 6 cycles of treatment without dose reduction. The high rate of treatment completion may explain the better response rate and survival outcomes in our study.

ICT also was a predictor of survival for locally advanced SCCHN. A recent meta-analysis study showed the ICT responders had better survival than nonresponders [28]. Moreover, a phase II study demonstrated that responders after one cycle of split-dose TPF ICT were a survival predictor for oral and oropharyngeal squamous cell carcinoma (OPSCC) [29]. A review article that recruited seven studies displayed that the standardized uptake value (SUV) reduction in interim PET scan may predict ICT response, PFS, and OS [30]. In our study, biweekly TPF ICT demonstrated around 90% RR and a high CR rate (31.0%). Also, the patients with CR had better OS and PFS than those with non-CR. Accordingly, patients with metabolic non-CR may need an additional treatment after ICT.

Limitations of our study include the lack of a comparator group and the relatively small sample size. Although the primary objective of this study is to investigate the response rate of ICT, the heterogeneous treatment during radiotherapy could have introduced major biases in the analysis of OS. Additionally, 14 of 54 (25.9%) patients could receive radiotherapy only, which raised the concern that the administration of ICT may compromise ﻿the completion of subsequent concomitant administration of chemotherapy or biotherapy. To draw definitive conclusions is difficult in this regard because compliance and toxicity data are differently reported. However, according to the Spanish TTCC trial, which used standard triweekly TPF regimen followed by concomitant CRT regimen, 30% of patients would never receive radiotherapy, 41% of patients needed dose reduction, and 17% of patients discontinued CRT treatment [7]. In our study, 4/58 (6.9%) patients did not receive the following radiotherapy, and 3/54 (5.6%) patient discontinued CRT treatment. The relative tolerability of the dose-dense TPF regimen may be attributed to fewer grade 3–4 adverse effects and shortened treatment duration. Additionally, because radiotherapy would follow the induction chemotherapy within 4 weeks, it is impossible to confirm our treatment response by repeat assessments that should be performed no less than 4 weeks after the RECIST criteria for the response classification was initially met. Finally, our study design is unlikely to answer the question of whether the combination of dose-dense induction therapy plus the following radiotherapy can beat conventional concomitant CRT therapy.

## Conclusions

In conclusion, the investigational regimen of biweekly TPF ICT appears to have favourable tolerability with an acceptable response rate compared to published studies. This regimen had fewer grade 3/4 hematologic adverse events including neutropenia, anemia, and thrombocytopenia than the triweekly TPF. Also, the survival figures were not inferior to the reported studies of triweekly TPF ICT. The patients with CR after ICT had superior survival outcomes than those with non-CR. Thus, future studies should focus on whether further treatments are necessary for patients with non-CR after ICT.

## Supplementary information

**Additional file 1: Table S1.** Multivariate analysis of overall survival and progression-free survival.

## Data Availability

The datasets generated and analyzed in the current study are available from the corresponding author upon reasonable request.

## Abbreviations

CI: Confidence interval
CR: Complete response
CRT: Chemoradiotherapy
CTVs: Clinical target volumes
ddTPF: Dose-dense TPF
EORTC: European Organization for Research and Treatment of Cancer
GTV: Gross tumor volume
HPV: Human papilloma virus
ICT: Induction chemotherapy
IMRT: Intensity-Modulated Radiotherapy
OS: Overall survival
PET: Positron emission tomography
PF: Cisplatin plus 5-fluorouracil
PFS: Progression-free survival
PR: Partial response
ROC: Receiver operating characteristic
RR: Response rate
RT: Radiotherapy
SCCHN: Squamous-cell carcinoma of the head and neck
SD: Stable disease
TPF: Cisplatin and 5-fluorouracil plus docetaxel
