# Supplementary material for: Dose-dense TPF induction chemotherapy for locally advanced head and neck cancer: a phase II study
Source: BMC Cancer. 2020 Sep 1;20:832. doi: 10.1186/s12885-020-07347-6 (PMC7465725; doi:10.1186/s12885-020-07347-6)
Supplement: Supplementary file 1 — Additional file 1: Table S1. Multivariate analysis of overall survival and progression-free survival. [file 12885_2020_7347_MOESM1_ESM.docx]

Table 3. Multivariate analysis of OS and PFS

| OS | | |
| --- | --- | --- |
| Variables | HR (95% CI) | *P* value |
| Age, ≤50 vs. >50 years | 0.503 (0.208–1.218) | 0. 128 |
| Stage, IVA vs. IVB | 1.769 (0.776–4.301) | 0.175 |
| Oropharynx vs. non-oropharynx | 2.425 (0.889–6.610) | 0.083 |
| Smoking, No vs. Yes | 1.611 (0.396–6.562) | 0.506 |
| Response rate, CR vs. PR | 4.250 (1.206–14.977) | 0.024 |
| CR vs. SD/PD | 17.196 (3.431–113.86.179) | 0.001 |
| PFS | | |
| Age, ≤50 vs. >50 years | 0.718 (0.340–1.516) | 0.385 |
| Stage, IVA vs. IVB | 1.558 (0.768–3.158) | 0.219 |
| Oropharynx vs. non-oropharynx | 1.418 (0.651–3.091) | 0.397 |
| Smoking, No vs. Yes | 0.934 (0.306–2.848) | 0.904 |
| Response rate, CR vs. PR | 3.199 (1.319–7.759) | 0.010 |
| CR vs. SD/PD | 10.159 (2.851–36.199) | <0.001 |

OS: overall survival; PFS: progression-free survival, CR: complete response, PR: partial response, SD: stable disease, PD: progressive disease
